# Use of tunica vaginalis graft for repair of traumatic bilateral testicular rupture after gunshot: a case report

**DOI:** 10.11604/pamj.2020.36.268.21988

**Published:** 2020-08-12

**Authors:** Faisal Ahmed, Mohammed Naji, Ahmed Al-Hitari, Qais Amer, Mohammed Al-sagheer, Umayir Chowdhury

**Affiliations:** 1 Urology Research Center, Al-Thora Hospital, Department of Urology, Ibb University of Medical Since, Ibb, Yemen; 2 Department of Urology, Dar Alshefa Hospital, Ibb, Yemen; 3 Department of Radiology, Dar Alshefa Hospital, Ibb, Yemen; 4 School of Medicine, Shiraz University of Medical Sciences, Shiraz, Iran

**Keywords:** Bilateral testis, gunshot, trauma

## Abstract

Bilateral testicular injuries are rare. However, the incidence of these injuries has been increasing in wartime. We describe the case of gunshot wound of the both testicle caused by high velocity bullets. The patient was managed by surgical exploration, debridement and repaired of both testis using tunica vaginalis. During the follow up, the left testis was not viable and there was a need for orchiectomy; in a follow-up of 4 months, ultrasonography showed a viable right testis with minimal atrophic change and the patient reported to have normal erection with borderline hormonal function. We describe this case of bilateral testicular rupture, which was repaired using tunica vaginalis as graft, with attention to the management and outcome of this injury process.

## Introduction

Bilateral testicular injury caused by gunshot is rare and represents proximally about 6-26% of all penetrating trauma. Recently, wider incidence of this injury has been noted in wartime [1,2]. Injuries of male genitalia could be very complex, and if not promptly treated, they lead to morbidity and mortality such as bleeding, decreased sexual function and infertility [3]. About 90% of high velocity bullets caused testicular explosion and orchiectomy may be necessary [4]. Severe testicular injury could affect the fertility and contribute to hypogonadism, which affects social confidence [5]. The aim of treatment is to save as much functioning of the testicular parenchyma as possible, anatomic and cosmetic preservation, and maintenance of sexual function [1]. We would like to describe a case of high velocity gunshot injury of both testicle and medial aspect of the left lower leg, which repaired Using tunica vaginalis as graft, with attention to the management and outcome of this injury process.

## Patient and observation

**Figure 1:**
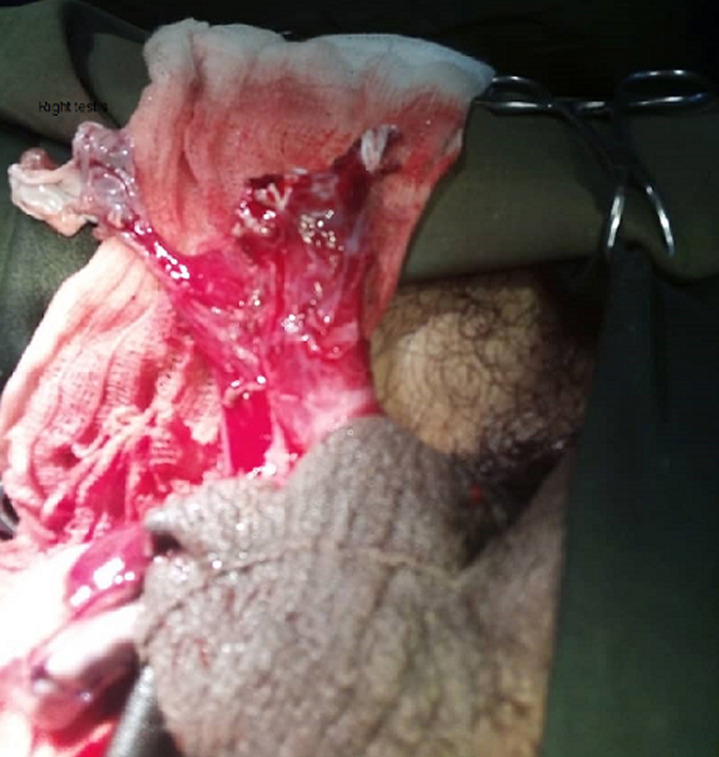
intraoperative photography showing complete damage to the right testis

**Figure 2:**
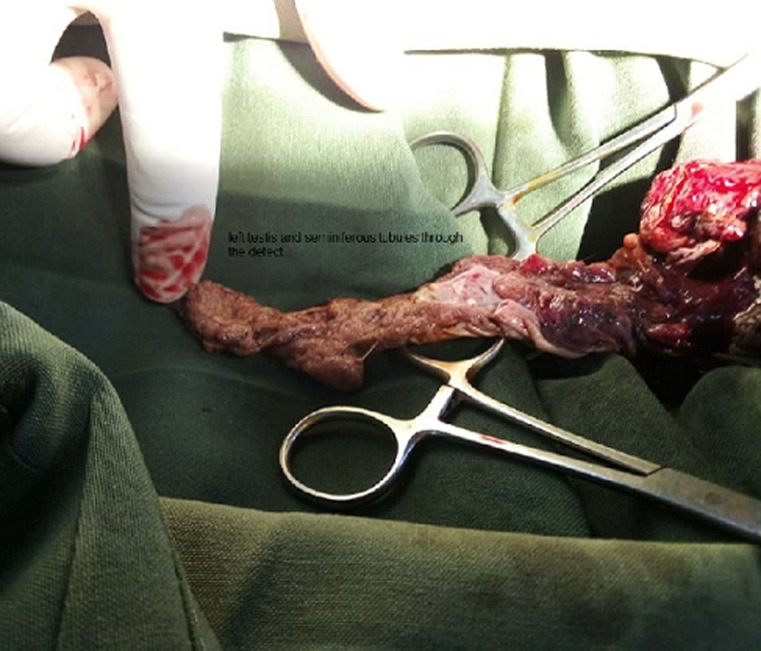
intraoperative photography showing complete damage to the left testis and seminiferous tubules through the defect (extruded seminiferous tubules)

A 20-year-old single male presented to the emergency department with scrotal rupture, left leg laceration and bleeding following a gunshot accident from a distance of 12-16 cm, which penetrated the scrotum and both testes and came out from the medial aspect of the left leg. The patient experienced severe pain in his scrotum and left lower extremity. Nothing significant was mentioned in his previous medical history. On arrival, he was noted to be conscious, and had no vomiting and no abdominal pain. In physical exam, his vital signs seemed normal (blood pressure: 120/75, pulse rate: 110 beats per minute), his abdomen was soft without tenderness, his scrotum had a big laceration, and both testes were completely ruptured (Figure 1, Figure 2). The exit wound was located in the medial aspect of the left knee. Other external genitalia were normal in inspection. He had no urethral meatus bleeding, but he had a laceration in the left leg. Blood profile tests such as complete blood cell, electrolyte profile, liver function test and coagulation test were normal; there was just a rise in WBC (white blood cell) 14000mg/dl. A focused assessment with ultrasonography for trauma (FAST) did not reveal any free fluid in the abdominal cavity. A plain film of the left leg showed a small fracture in the talus bone. After resuscitation, the patient was transferred to the operation room for urgent scrotal exploration. The patient was informed that an orchiectomy might be required if his testis cannot be repaired. After that, emergency scrotal exploration was done; about 200ml of the hematoma was evacuated, tunica vaginalis and both testes were exposed and completely damaged, and some gunshot fragment was identified and removed. Then, the scrotum was irrigated with 3000ml of normal saline.

**Figure 3:**
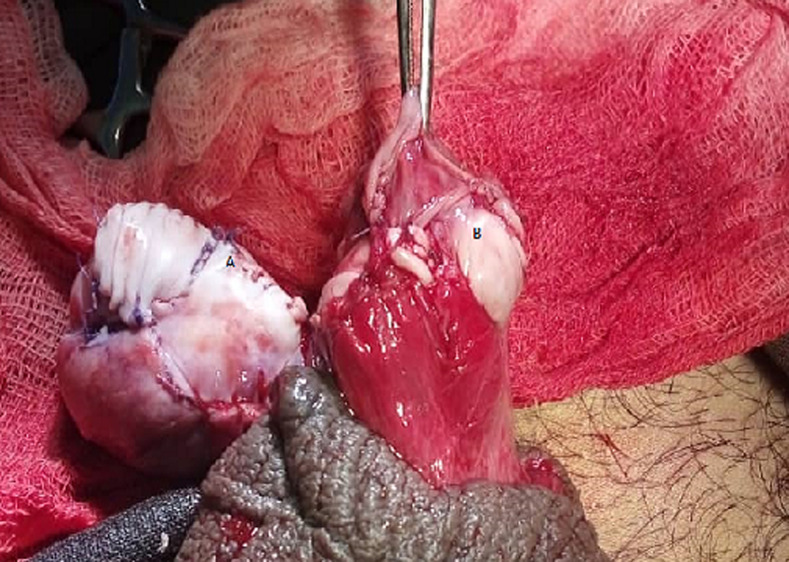
intraoperative photography showing the repaired testes A) left testis; B) right testis

**Figure 4:**
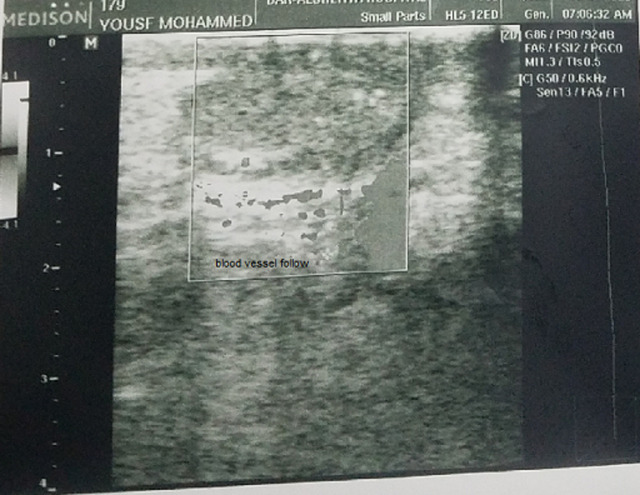
ultrasonography of the right testis showing a normal right testis with normal blood vessel follow and minimal atrophic change 4 months after the procedure

Debridement of necrotic testicular tissue was subsequently preformed. However, the ruptured testis could not be closed primarily because the extruded seminiferous tubules and the big tissue defect of the tunica albuginea did not allow the apposition of the tunica albugineal edges. Therefore, closure of the tunica albuginea using tunica vaginalis as a graft tissue was done (the free edges of the parietal tunica vaginalis was used to create neocapsulae around the testis parenchymal tissue) by using Vicryl suture bilaterally, and the drain was left in situ (Figure 3). Skin was closed with nylon 3/0. It should be mentioned that vas deference and epididymis were damaged, and we recommended the delayed repair of vas deference and epididymis with microsurgical procedure. Finally, Foley catheter Fr 16 was inserted. The laceration of the left leg was irrigated with 2000ml of normal saline and repaired by an orthoptist. During the same admission, the patient received ceftriaxone 2 gram daily and was under the joint care by the orthopedist. The drain and catheter were removed three-days post-operatively. The patient was discharged one-week later with outpatient follow up. Additionally, a three-week course of cephalexin and ibuprofen was recommended for the patient. Unfortunately, during post-operative period there were some complications in orthopedic and urologic procedures, such as fever and discharge from the site of operation. Additionally, the left testis was not viable and there was a need for orchiectomy during the follow up of one week. Follow-up Doppler ultrasonography at 4 months showed a viable right testis with minimal atrophic change (Figure 4). Four months after the surgery, the patient had normal erection and the anorchide serum hormone concentration such as (serum testosterone level was 6.27ng/ml, follicle-stimulating hormone (FSH) 120.3nl/ml, luteinizing hormone (LH) 63.69nl/ml and the prolactin level was 11.05ng/ml), which show that the patient had borderline normal hormonal profile. The semen analysis showed the total sperm count: 12 million, volume: 2.5cc, motility: nonviable and normal forms: 70% and the patient was happy with the cosmetic results.

**Ethical consideration:** the patient gave their written informed consent for participation in our study.

## Discussion

The incidence of penetrating testicular injuries has been on increase over the years, due to war and violence. Indeed, bilateral testicular injuries are more common in penetrating trauma and the common cause of these injuries is gunshot [6]. Early surgical exploration in scrotal gunshot injuries is recommended to save the damage to the testis, perform debridement of nonviable testis, drain the scrotal hematoma and control bleeding. It was reported that 80% of the ruptured testis could be saved if surgical repair is preformed within 72 hours of testicular trauma [7]. Reconstruction of the testis may not be useful for sperm production because of association with ductal obstruction, but it is important for preservation of hormonal production and cosmetic appearance. Additionally, repair of epididymis and vas deference should be managed by delayed microsurgical repair [8,9]. In high velocity bullets, due to testicular explosion, it is impossible to save the testicular tissue and in 90% of cases, orchedioctomy may be mandatory [10]. In our case, we tried to save both testes, but unfortunately due to the nonviable left testis tissue, the left testis was exposed again and orchiectomy was mandatory after one week of follow-up period.

In cases of complete testis amputation, a microsurgical measure should be taken for the best chance of viable reanastomosis [9]. However, in our center, a microsurgical specialist was not available and reanastomosis was done by a general urologist. If a large defect is observed during the surgery, grafts consisting of dermis, elastic material or tunica vaginalis may be useful [10]. Previous literature demonstrated that tunica vaginalis graft showed a better outcome than synthetic graft [7]. In our case, we used tunica vaginalis as the graft to repair the large defect. About 6% of patients who had experienced gunshot injuries in the testis had erectile dysfunction [4]. In our case, the patient was satisfied with the surgery and had erectile function without pain. It should be mentioned that to fertility and sexual function are potentially damaged when bilateral testicular injuries occur and there is no correlation between the type of treatment and the degree of testicular function after surgery. Furthermore, the type of injuries might be the primary determinant of the outcome in these cases [10]. In our cases, the patient reported a good erection, but his semen profile was damaged.

## Conclusion

Emergency assessment, diagnosis, and early scrotal exploration are important for treatment of testicular rupture and using the tunica vaginalis as graft for repaired ruptured testis. Additionally, we did look at whether the patient was able to preserve the function of his testis by assessing erectile function following this event.

## References

[1] Ficarra V, Caleffi G, Mofferdin A, Zanon G, Tallarigo C, Malossini G (1999). Penetrating Trauma to the Scrotumand the Corpora cavernosa Caused by Gunshot. Urol Int.

[2] Salvatierra O, Rigdon WO, Norris DM, Brady TW (1969). Vietnam experience with 252 urological war injuries. J Urol.

[3] Jolly B, Sharma SK, Vaidyanathan S, Mandal A (1994). Gunshot wounds of the male external genitalia. Urol Int.

[4] Monga M, Moreno T, Hellstrom WJ (1995). Gunshot wounds to the male genitalia. J Trauma Acute Care Surg.

[5] Addas F, Yan S, Hadjipavlou M, Gonsalves M, Sabbagh S (2018). Testicular Rupture or Testicular Fracture? A Case Report and Literature Review. Case Rep Urol.

[6] Bhatt S, Dogra VS (2008). Role of US in testicular and scrotal trauma. Radiographics.

[7] Ferguson GG, Brandes SB (2007). Gunshot wound injury of the testis: the use of tunica vaginalis and polytetrafluoroethylene grafts for reconstruction. J Urol.

[8] Haddon Jr W (1980). Advances in the epidemiology of injuries as a basis for public policy. Public health reports.

[9] Furr J, Culkin D (2017). Injury to the male external genitalia: a comprehensive review. Int Urol Nephrol.

[10] Cass A, Ferrara L, Wolpert J, Lee J (1988). Bilateral testicular injury from external trauma. J Urol.

